# Comparative Analysis of Clinical Effects of Mineral Trioxide Aggregate in the Treatment of Endodontic Diseases

**Published:** 2019-04

**Authors:** Jian SUN, Yuanyuan WEI, Xiaobei TIAN, Qinglin MEN

**Affiliations:** 1. Department of Endodontics, Xuzhou Stomatological Hospital, Xuzhou 221003, P.R. China; 2. Department of Prosthodontics, Xuzhou Stomatological Hospital, Xuzhou 221003, P.R. China; 3. Teaching and Research Office of Stomatology, Xuzhou Medical University, Xuzhou 221003, P.R. China

**Keywords:** Oral biomaterials, Mineral trioxide aggregate (MTA), Root canals, Endodontic disease

## Abstract

**Background:**

We aimed to evaluate the efficacy of mineral trioxide aggregate (MTA) in the treatment of endodontic disease.

**Methods:**

Oversell, 384 patients with endodontic disease treated in Xuzhou Stomatological Hospital, Xuzhou, China, from June 2015 to June 2017 were selected, and randomly divided into four groups with 96 cases per group. The repair effects of MTA, zinc phosphate cement (ZPC), silver amalgam and light-curing calcium hydroxide (LCH) on the teeth and dental pulp of patients in the four groups were compared. Meanwhile, the ill symptoms of the patients were observed to confirm whether they could be alleviated. Besides, whether the repair effects were related to ages of patients, perforation diameters of diseased teeth and repair materials was discussed.

**Results:**

The success rates of MTA group, ZPC group, LCH group and silver amalgam group were 90.6%, 68.7%, 70.8% and 52.1%, respectively. The success rate of MTA group was significantly higher than that of ZPC group, silver amalgam group and LCH group. When the success rates of four groups were compared, the differences were statistically significant (*P*=0.0072). The patient's age, repair material and perforation diameter were positively correlated with MTA repair effect (*P*=0.003, *P*=0.002, *P*=0.01). The patients' teeth in each group were repaired with different materials, and the reexamination was conducted 4 weeks later. Three patients in the silver amalgam group were found to have gingival swelling.

**Conclusion:**

The therapeutic effect of MTA was significant in the treatment of endodontic disease, and it is worthy of clinical application.

## Introduction

Endodontic disease is a kind of clinically common dental disease, mainly including caries, periapical disease, tooth hypersensitivity, pulp inflammation, necrosis and metamorphosis, etc. Patients will suffer from varying degrees of pain, black teeth and broken teeth, which have different influences on the patients’ appearance, normal diet and life quality. Endodontic disease can be caused by multiple factors, such as physical and chemical stimulation, trauma, immune response and bacterial infection, in which bacterial infection is a key factor in the pathogenesis of endodontic disease (1).

Mineral trioxide aggregate (MTA) is hydrophilic, mainly composed of tricalcium silicate, tricalcium aluminate and other elements, and has durable closeness, good tissue compatibility and other capabilities. Therefore, it has already been widely used in apexification, direct pulp capping, retrograde filling, perforation and repair of pulp floor and other fields in the treatment of endodontic disease.

We intended to observe the therapeutic effects of MTA, zinc phosphate cement (ZPC), light-curing calcium hydroxide (LCH) and silver amalgam in the repair of pulp floor and root canal perforation, and compare the repair effects of the four methods on different pathological types of perforation.

## Materials and Methods

### General information

Overall, 384 patients with endodontic disease treated in Xuzhou Stomatological Hospital (Xuzhou, China) from June 2015 to June 2017 and confirmed by imageological diagnosis and surgical exploration were selected, in which there were 190 males and 194 females aged between 18 yr old and 80 yr old.

Patients and their families were informed in advance of the study, and singed an informed consent form. This study was approved by the Ethical Committee of Xuzhou Stomatological Hospital (Xuzhou, China).

### Inclusion and exclusion criteria

Inclusion criteria: Patients with good compliance, pulpless teeth caused by pulp floor damage due to iatrogenic root canal perforation, perforation of pulp floor and caries lesion; diseased teeth with or without fistula; patients suffering from red and swollen periodontal tissue and percussion pain, without periodontal disease; patients with tooth mobility ≤ I°; patients without acute or chronic systematic disease; patients with dental crown damage <1/2; patients signing informed consent.

Exclusion criteria: patients with serious facial and jaw trauma, dental caries, diffuse periodontitis or hypertension.

### Materials

MTA (Dentsply, USA), light-cured glass ionomer cement (Shanghai Dental Materials Factory), silver amalgam (AT&M Biomaterials Co., Ltd.), light-curing calcium hydroxide (DMG, Germany).

### Methods

Under local anesthesia, perforation and repair were made with saliva isolation by rubber dam. As to iatrogenic pulp floor perforation, perforated parts of the diseased teeth were washed with normal saline and hydrogen peroxide in suitable concentration. After several times, bleeding was stopped by iodine cotton ball. The perforated sites were repaired with MTA after they became completely dry. As to pulp floor perforation caused by dental caries, caries lesion and pulp floor inflammatory granulation tissues were firstly removed, and then the perforated sites were washed repeatedly and alternatively by normal saline and sodium hypochlorite.

Repair and perforation were made with MTA after bleeding was stopped and dried (1). The use of MTA should be in strict accordance with instructions. Firstly moderate MTA was mixed with distilled water to reach slightly moistened state, and then the MTA under slightly moistened state was placed into the perforated sites of the patients, pushed and pressed slightly. After the correct position was confirmed by X-ray films, it was compacted and solidified for 3–4 hours. After filling, a small wet iodine cotton ball was placed into dental pulp cavity of the patients and closed temporarily with sealing label for 2 days. After 5∼7 days, return visit was made. When the perforated site was close to the entrance of the root canal, the root canals of the patients were firstly inserted by enlarge needle, in order to avoid the access to root canals at the time of MTA repair, and then result in the block of root canals and the failure of the treatment. At the time of return visit, the temporary sealing materials were removed. After being rebased by carboxylic acid zinc, permanent filling was done with 3M Z250 light-cured resin.

The other three groups of operations were the same as above steps. Return visits were done on the next day. Good repair effect was confirmed by X-ray films, and whether MTA, ZPC, silver amalgam and LCH had been hardened completely or not should be confirmed. After 1 week of observation, no symptoms appeared, and the dental pulp cavity was filled by light-cured resin or silver amalgam. Return visits were done after 4 weeks, in order to check whether there were adverse reactions. Review should be made after 1 year, in order to check whether there was recurrence. The above operations were made and finished by one doctor.

### Efficacy evaluation

Evaluation criteria for clinical efficacy: 1) Success: no subjective symptom after treatment; the diseased teeth are not loosened or are improved in loosening in clinical examination; patients have no percussion pain, occlusion pain, red and swollen gingiva or pressing pain; the filling is complete; there is no periodontal pocket; masticatory function is fine. It is shown that new hard tissues at perforated places form compact image or the sparse areas at perforated places of original pulp floor shrink or disappear by X-ray examination, and the lesion at the place of original broken root tip is still or shrinks. 2) Failure: there is still spontaneous pain; there is percussion or bite discomfort; the filling falls off; the looseness of diseased teeth is not improved or aggravated; there is periodontal pocket or sinus tract; abscess is formed; gingivae become red and swollen; it is shown that the sparse areas at perforated places of pulp floor expand, or the shadow at the place of secondary root tip expands; if any one of above items appears, that means failure. The curative effects on diseased teeth with perforated diameter <2mm and ≥2mm were recorded, and statistics was made.

### Statistical methods

Statistical Product and Service Solutions Version 22.0 (Chicago, IL, USA) was used in the processing of all data; measurement data were expressed as mean ± standard deviation; *t* test was used for comparisons of normally distributed data among groups; abnormally distributed data were expressed as the median (interquartile range); and the nonparametric rank sum test was used in comparisons among groups. Analysis of variance was used in mean comparisons among groups; χ^2^ test was used for the comparison of ratio; Logistic regression was used in the analysis of relative factors; the test level α=0.05; *p*<0.05 suggested that differences were statistically significant.

## Results

### Basic information

A total of 384 clinical patients were included, in which there were 134 cases of iatrogenic root canal perforation, 121 cases of deep caries root canal perforation and 129 cases of pulp floor perforation. The differences were not statistically significant in the comparisons of ages, gender constitute ratios, diseased teeth types, pathological types and perforated diameters among groups (*P*=0.728, *P*=0.987, *P*=0.976, *P*=0.252, *P*=0.981), and the data were comparable (Table 1).

**Table 1:** Comparisons of general data in each group of research objects

***Group***	***MTA***	***ZPC***	***Silver amalgam***	***LCH***	***Statistic***	***P***
Gender					*χ^2^*=0.142	0.984
Male	50	47	45	48		
Female	46	49	51	48		
Age (yr)	38.76±5.89	37.54±6.43	36.38±6.74	38.52±5.48	*t*=0.68	0.728
Type of diseased teeth					χ^2^=0.186	0.976
Premolar teeth	26	27	25	28		
Molar teeth	96	96	96	96		
Pathological type					χ^2^=0.252	0.252
Deep caries root canal perforation	30	32	28	31		
Iatrogenic root canal perforation	34	33	31	36		
Perforation of pulp floor	32	31	37	29		
Perforated diameter					χ^2^=0.172	0.981
<2mm	52	51	54	56		
>2mm	44	45	42	40		

### Comparisons of curative effects among groups

The success rates of MTA group, ZPC group, LCH group and silver amalgam group were respectively 90.6%, 68.7%, 70.8% and 52.1%; the success rate of MTA group was significantly higher than that of ZPC group, silver amalgam group and LCH group; and the differences were statistically significant (*P*=0.0072) in the comparisons of four groups of success rates (Table 2). The differences were statistically significant in the comparisons between MTA group and ZPC, LCH and silver amalgam groups (χ^2^=9.28, 9.68, 11.86, *P*=0.0024, 0.0023, 0.0012). The difference was not statistically significant in the comparison between ZPC group and LCH group (χ^2^=1.14, *p*=0.56). The differences were statistically significant in the comparisons between ZPC group and silver amalgam group and between LCH group and silver amalgam group (χ^2^=9.28, 9.68, 11.86, *P*=0.0084, 0.0079).

**Table 2:** Efficacy comparisons of dental restoration among groups

***Group***	***MTA***	***ZPC***	***LCH***	***Silver amalgam***	***Statistic***	***P***
Total	96	96	96	96		
Successful case	87	66	68	50		
Failed case	9	30	29	46		
Success rate	90.60%	68.70%	70.80%	52.10%	χ^2^=9.84	0.0072

### Relative factor analysis on the effects of MTA in repair and perforation

The ages, repair materials and perforated diameters were positively correlated with the effects of MTA in repair (*P*=0.003, *P*=0.002, *P*=0) (Table 3).

**Table 3:** Relative factor analysis on the effects of MTA in repair and perforation

***Variable***	***β***	***t***	***P***
Gender	0.091	0.258	0.119
Age (yr)	0.748	4.859	0.003
Diseased teeth type	0.114	1.914	0.182
Repair material	0.641	5.232	0.002
Perforated diameter	0.202	8.278	0.001
Pathological type	0	0.121	0.112

### Adverse reaction

Each group of patients' teeth was respectively repaired with different materials. Reviews were done after 4 weeks. The gingiva of 3 cases of patients became red and swollen in silver amalgam group and had been improved after symptomatic treatment. There were no adverse reactions in other three groups.

## Discussion

Endodontic disease has a higher and higher incidence, and has been one of the three major diseases to be prevented and treated for human being due to the changes of dietary habits and living habits as well as no attention to personal hygiene. Dental pulp and alveolar socket damage or dental caries, inflammatory reaction of periodontal tissue or root canal perforation (2) not only bring different degrees of pain to patients, but also affect normal diets of patients.

Therefore, repairing perforated sites and eliminating inflammatory reaction is the key to cure endodontic disease (3). There are many materials to repair perforation, for example, MTA, ZPC, LCH and silver amalgam. Silver amalgam is a kind of very traditional material to repair perforation. LCH has better biocompatibility and certain bacteriostasis, and can induce periodontal tissue to form calcium compound. It has better compression resistance capacity as well as good cohesive force after LCH solidification. It is unnecessary to be mixed and relatively convenient to be used (4).

ZPC has good cohesion, biocompatibility, release of fluoride and resistance to solubility, as well as good hydrophilia. MTA is clinically the most commonly used perforation repair material (5) favored by doctors, and has been approved by Food and Drug Administration (FDA) (6). It has good histocompatibility and durable closeness, and can also induce tissue regeneration.

The solidification of MTA is not affected by humid environment and whether the blood pollution exists or not. It has lower solubility, better stress tolerance and better ray resistance, which is easy to be identified (7–12) in the X-ray film because it is higher than dentin, and it is in favor of subsequent visits and judgment of repair efficiency. In addition, MTA also plays the role of accelerating the growth of periodontal bone and bone regeneration (13), and does not have neurotoxicity and mutagenicity (14, 15). How to retain diseased teeth to the upmost extent to improve life quality of patients is one of the key issues to be considered. It is indispensable (16) for the repair and treatment of root canal perforation and pulp floor perforation to choose superior repair materials. Whichever filling material is used, it is necessary to ensure that the filling material is sterile (17). Therefore, this paper adopted MTA, ZPC, silver amalgam and LCH to repair pulp floor perforation and root canal perforation, and compared the perforation and repair effects of the four materials.

In this study, the results showed that the success rate of MTA group was significantly higher than that of the other three groups, which was closely related to the features of MTA. The pH value of MTA is weakly-alkaline with a certain antibacterial effect. MTA still has good closeness even in blood pollution environment, and can induce tissue regeneration. Micro-leakage happens rarely even if MTA gets solidified in a humid environment. This is the feature that the other three materials do not possess (18).

Relative factors of the effect of MTA in repair and perforation were analyzed, and the results showed that repair efficiency of MTA was positively correlated with the ages of patients, repair materials and perforation diameters. The curative effect of MTA in perforation repair was related to the size of perforated diameter, and was consistent with the results that the larger the perfo-rated diameter, the worse the prognosis result (19). But it had nothing to do with gender, pathological type and type of diseased teeth. In addition, the effect of MTA in perforation repair was also associated with precipitating factors of perforation. According to clinical statistics, the effect of MTA on cariogenic perforation patients was poorer, and the effect of MTA on iatrogenic perforation patients was also poorer (20). Pulp floor perforations of the patients were repaired with MTA. 2 years later, it was found that the pain and swelling of diseased teeth disappeared and functions were kept stable (21) at the time of return visits. Red and swollen gingivae appeared in silver amalgam group. This was because the tissue under the perforation was stimulated to produce inflammatory reaction due to compression when silver amalgam was filled, and there was no effective combination between silver amalgam and dentin, so micro-leakage was more serious (22).

## Conclusion

MTA is significantly better than ZPC, LCH and silver amalgam in the treatment of endodontic disease, so it can be widely used in clinical practice.

## Ethical considerations

Ethical issues (Including plagiarism, informed consent, misconduct, data fabrication and/or falsification, double publication and/or submission, redundancy, etc.) have been completely observed by the authors.
